# Psychosocial and Financial Burden of Therapy in USA Patients with Pulmonary Arterial Hypertension

**DOI:** 10.3390/diseases8020022

**Published:** 2020-06-13

**Authors:** Scott A. Helgeson, Divya Menon, Haytham Helmi, Charitha Vadlamudi, John E. Moss, Tonya K. Zeiger, Charles D. Burger

**Affiliations:** 1 Department of Pulmonary Medicine, Mayo Clinic, Jacksonville, FL 32224, USA; Charitha.Vadlamudi@bmc.org (C.V.); moss.john@mayo.edu (J.E.M.); zeiger.tonya@mayo.edu (T.K.Z.); burger.charles@mayo.edu (C.D.B.); 2 Department of Pulmonary and Critical Care Medicine, Tufts Medical Center, Boston, MA 02111, USA; dmenon@tuftsmedicalcenter.org; 3 Department of Emergency Medicine, University of Florida, Jacksonville, FL 32224, USA; Haytham.Helmi@jax.ufl.edu

**Keywords:** pulmonary hypertension, psychosocial, financial, treatment

## Abstract

Pulmonary arterial hypertension (PAH) is a devastating disease with significant morbidity and mortality. There are many psychosocial and financial implications of this disease; however, little is known how this affects the treatment of PAH patients. A questionnaire-based prospective cohort study was performed on 106 PAH patients from a Pulmonary Hypertension Center and the Pulmonary Hypertension Association national conference in 2018. The demographic, treatment, psychosocial, employment, financial impact on treatment data was obtained. The majority of patients had cardiopulmonary symptoms despite treatment. The symptoms affected their social and work lives, with about one in three applying for disability because of their PAH. The majority of PAH patients had insurance coverage, but still noted a significant financial burden of the disease, with nearly a half who needed financial assistance to pay for their PAH medications. Thirty (28.3%; 95% CI, 20.6–37.5%) patients mentioned they changed their medication regimen, with some skipping doses outright (28 [26.4%; 95% CI, 19–35.6%]) in order to save money. PAH continues to cause significant psychosocial and financial burden on patients despite advances in medications. This impact ranged from dissatisfaction with quality of life, to unemployment, to altering their medication regimen to save money.

## 1. Introduction

Pulmonary arterial hypertension (PAH) is a progressive disease of the lung vasculature that causes significant morbidity and mortality if not adequately treated. It is a rare disease that has an estimated prevalence in the United States (US) of 451 per million in people 65 and older and 109 per million in people younger than 65 years old [1]. Diagnosis of PAH is focused on confirming elevated pressures in the pulmonary vasculature while simultaneously trying to determine an underlying cause.

Well-recognized symptoms of PAH include dyspnea, chest discomfort, palpitations, lightheadedness, and peripheral edema. Treatment for PAH attempts to decrease the pulmonary pressures and vasculature remodeling by using a combination of phosphodiesterase-5 inhibitors, endothelin receptor antagonists, soluble guanylate cyclase stimulants, and prostacyclin receptor agonists. These medications may also contribute to symptom burden as flushing, headache, nasal stuffiness, nausea, diarrhea, and chronic pain, which are all common side effects. These symptoms often affect the patient’s psychosocial quality of life, even on treatment, which has been previously demonstrated [2,3,4,5,6,7,8,9]. These prior studies have also shown significant impacts on the physical, emotional, and social aspects of not only a patient’s life, but also on their caregivers. They also show that many of the burdens persist despite appropriate treatment.

Forty years ago, the median survival of PAH patients was 2.8 years, but treatment advances have improved the 5-year survival to 65% [10,11]. Nonetheless, newer medications are costly and are associated with a significant economic burden [12,13]. Multiple studies have shown the average monthly costs for PAH patients to be between USD 2023 and USD 9925 [1,14,15,16].

While the cost has been quantified, there are limited data on the direct impact to the patient’s quality of life, financial status, or medical adherence. The current study utilized questionnaire responses to describe the patient’s perspective of that impact.

## 2. Material and Methods

A questionnaire-based prospective cohort study was performed on adult patients with World Health Organization (WHO) Group 1 PAH currently undergoing therapy in two separate patient populations. The two patient populations were a consecutive group of PAH patients from the Pulmonary Hypertension (PH) Clinic at Mayo Clinic Florida (MCF) (March through June 2018) and a second group of volunteers that randomly visited the research room at the 2018 Pulmonary Hypertension Association (PHA) International PH Conference (June 29–July 1 2018, Orlando, Florida). A 25-point questionnaire was created and included questions on basic demographic data, comorbidities, medication regimens, quality of life, compliance with medications and follow-up, and economic and social challenges of medication regimens, including insurance and out-of-pocket data (Supplement Table S1). Exclusion criteria included pediatric (age < 18 years) PAH patients, patients with prior lung transplantation, absence of PAH therapy, and clinical trial participants. All patients gave their informed consent for inclusion before they answered the questionnaire. Data was de-identified upon collection. The study was conducted in accordance with the Declaration of Helsinki and the study was approved by the Mayo Clinic Institutional Review Board (17-006718) and Research Committee for the PHA (PHA-18/106–351).

At MCF, the questionnaire was administered either in-person during a PH clinic visit or by telephone after the visit to consecutive patients with an established Group 1 PAH diagnosis. At PHA, group 1 PAH patients who volunteered to participate in the PHA’s research room during the national conference, completed the same questionnaire. There were no duplicate patients. The two cohorts were compared to each other only to show differences in demographics and treatment data.

Statistical analysis was performed using JMP^®^ 14.1.0 (SAS Institute Inc., Cary, NC, USA). Continuous data were analyzed by a 2-sample Wilcoxon rank sum test. Categorical data were compared using a Pearson χ^2^ or Fisher exact test, where appropriate depending on the number of patients in each analysis. A *p* value less than 0.05 was considered significant.

## 3. Results

A total of 106 patients participated in this study with 57 patients in the MCF group and 49 patients in the PHA group, with demographic data shown in Table 1. Patients in the MCF group were older and more likely to have diagnostic group 1.1, 1.2, and 1.3 PAH. The large majority of MCF patients lived in the southeast US, whereas less than one-third of the PHA group were from the southeast and represented a wider distribution of residence throughout the US.

The medication regimen was similar between the two groups, with the majority of patients on combination therapy (75.4%) (Table 2). There was a trend for more patients to be on riociguat and treprostinil infusion from the PHA group and more calcium channel monotherapy for vasoreactive PAH in the MCF patients.

### 3.1. Symptoms

**Figure 1:**
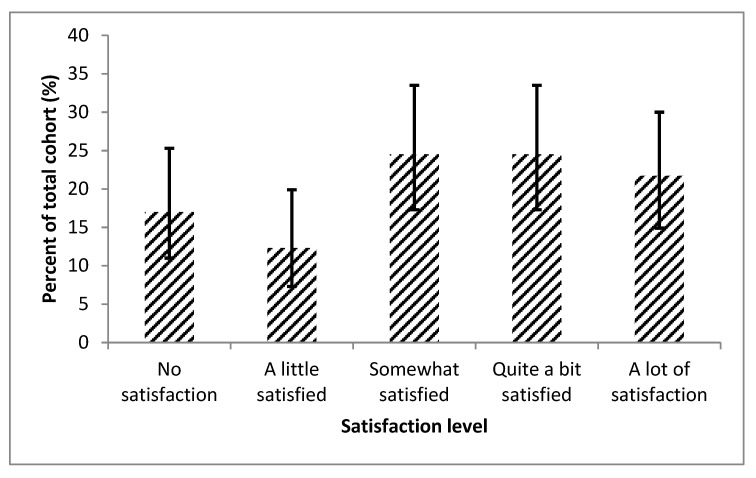
This figure displays the entire cohort’s satisfaction with their current level of social activity, displayed with 95% confidence intervals. This figure shows that despite treatment more than half of the patients were only somewhat satisfied or worse with their social life.

**Figure 2:**
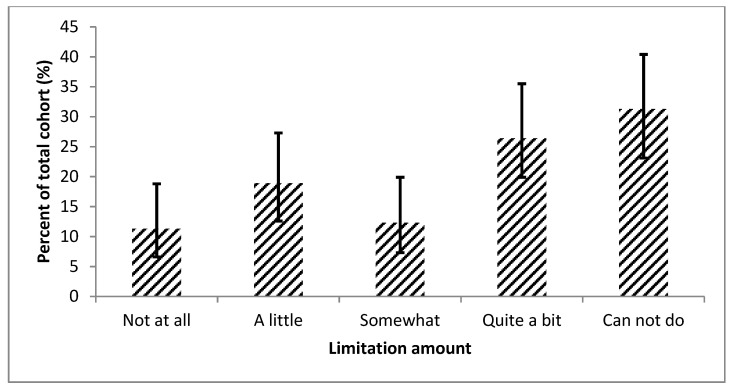
This figure displays how much the entire cohort’s current health status limits them in performing 2 h of physical activity, displayed with 95% confidence intervals. This figure shows that nearly two out of three patients present some or worse limitations when performing 2 h of physical activity.

The majority of patients (90 [84.9%; 95% CI, 76.9–90.5%]) still had cardiopulmonary symptoms (shortness of breath, chest pain, lightheadedness with exertion, ankle swelling, or palpitations) despite current medication regimen. More than half of the patients (57 [53.8%; 95% CI, 44.3–63.0%]) were only somewhat satisfied or worse with their social life (Figure 1). Over two-thirds of the patients (75 [70.1%; 95% CI, 61.5–78.6%]) had some or worse limitations with two hours of physical activity (Figure 2).

### 3.2. Employment

Of the 66 patients who were employed when initially diagnosed with PAH, 35 (53.0%; 95% CI, 41.2–64.6%) patients were no longer working. Of those currently employed, 11 patients (35.5%; 95% CI, 21.1–53.1%) worked less than 30 h a week. About one-third of patients (12 [38.7%; 95% CI, 23.7–56.2%]) that were currently employed missed more than 10 days of work the prior year because of their disease. Forty-one (39.0%; 95% CI, 30.3–48.6%) patients applied for disability benefits because of their PAH diagnosis.

### 3.3. Insurance Coverage

**Figure 3:**
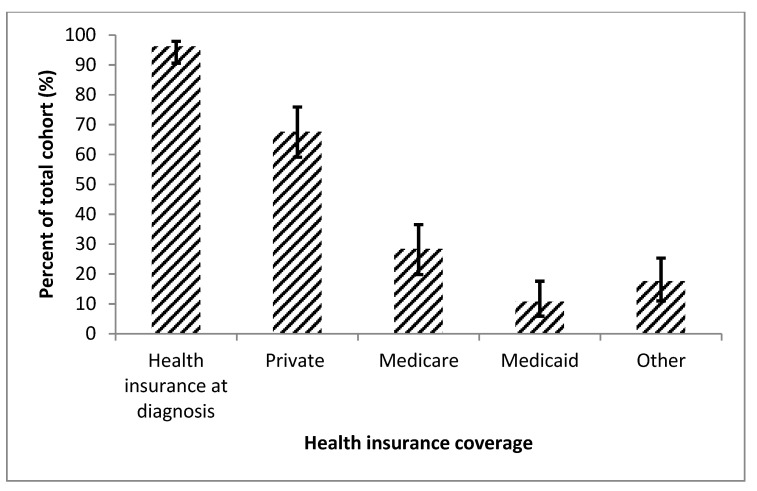
This figure shows the entire cohort’s insurance coverage at time of diagnosis (patients with double coverage = 25 [23.6%]), displayed with 95% confidence intervals. This figure shows that nearly all the patients had insurance patients, with the most common coverage type being private insurance.

In the entire cohort, 102 (96.2%; 95% CI, 90.7–98.5%) patients had insurance coverage at the time of diagnosis (Figure 3). The majority had private insurance (69 patients [67.6%; 95% CI, 58.1–75.9%]). Medicaid was the least common health insurance with only 11 patients (10.8%; 95% CI, 6.1–18.3%). Of the patients who had health insurance, 14 (13.7%; 95% CI, 8.4–21.7%) stated that the insurance did not fully cover the expenses associated with their PAH diagnosis.

### 3.4. Financial Burden

In the entire cohort, 87 (82.1%; 95% CI, 73.7–88.2%) patients had out-of-pocket expenses associated with their PAH diagnosis. Approximately one-third (33 patients [31.1%; 95% CI, 23.1–40.5%) had out-of-pocket expenses of at least USD 1,000 per month. Even with the patients with less out-of-pocket expenses, nearly one-half (33 patients [37.9%; 95% CI, 28.5–48.4%]) felt the expense burdensome.

**Figure 4:**
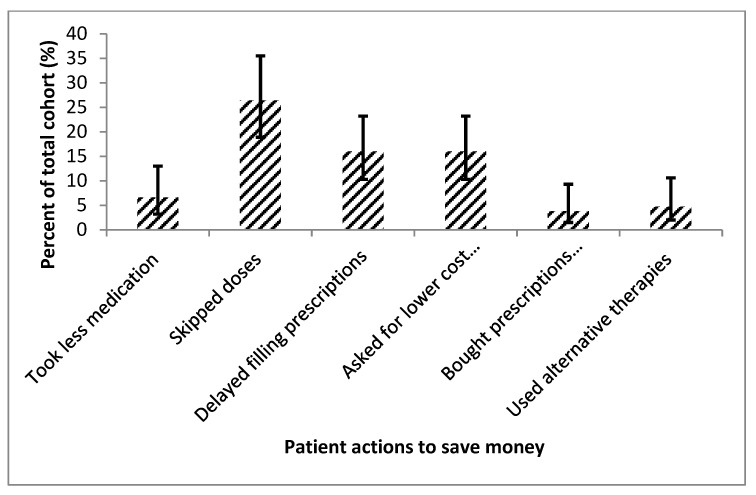
This figure shows the entire cohort’s difficulties with therapy compliance due to a financial burden, displayed with 95% confidence intervals. This shows that many of the patients attempted many cost-coping strategies.

**Figure 5:**
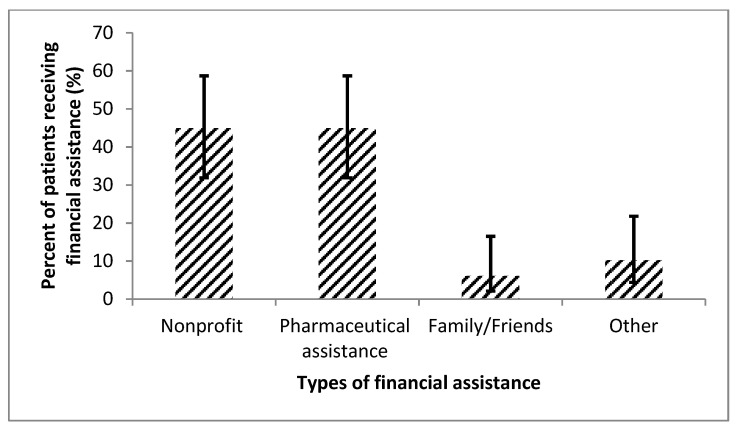
This figure shows the types of external financial assistance (*n* = 49 [46.2%]), displayed with 95% confidence intervals. Nonprofit organizations and pharmaceutical assistance helped support nearly all the patients who required external financial assistance.

Patients endorsed multiple challenges with compliance resulting in cost-coping strategies as detailed in Figure 4. Thirty (28.3%; 95% CI, 20.6–37.5%) patients mentioned they changed their medication regimen, with some skipping doses outright (28 [26.4%; 95% CI, 19.0–35.6%]) in order to save money (Table 3). Of those 28 patients who skipped doses to save, 10 (35.7%; 95% CI, 20.7–54.1%) patients missed medications weekly or more. About half of the patients had some type of financial assistance to support them with their PAH diagnosis, with the majority (22 patients [44.9%; 95% CI, 31.9–58.7%]) having a nonprofit organization provide support (Figure 5).

## 4. Discussion

This study focused on patient reported economic and psychosocial implications of PAH therapy in two different patient cohorts of established group 1 PAH patients, one from Mayo Clinic Florida and the second from the research room at the 2018 PHA meeting. While there were some minor demographic differences, most were women and on combination PAH therapy.

The present study is consistent with previously published studies regarding patient’s quality of life with PAH despite treatment [2,3,4,5,6,7,8,9]. Prior studies have shown that the physical, emotional, and social realms of patients were all severely decreased in patients diagnosed with PAH despite appropriate treatment. Certain subtypes of PAH, such as scleroderma associated, typically have worse quality of life scores [9]. Other studies show that patients on intravenous therapy have worse scores [4,8,9]. One study done in the UK showed that appropriate treatment improved their quality of life “a lot” [17]. Another study done in Portugal also showed that the significant quality of life disturbances associated with PAH was not just in the USA [18]. Despite studying quality of life in these patients, only the present study assessed the patient’s employment disability.

In addition, other published studies have focused on the monetary impact of PAH therapy [12,13,14,15,16]. These studies all show there are significantly increased costs associated with PAH, whether it be from physician visits, medications, or hospital admissions. An economic benefit has been shown by starting combination therapy early with increased pharmaceutical costs offset by reduced hospital admissions [12]. Despite this economic advantage, there is large impact on the patients with possible pharmaceutical costs approximately USD 2,023 to USD 11,875 per month [1,15,16,19]. A recent systematic review found that there were no studies evaluating indirect costs to PAH, such as employment loss and disability [19].

Our study also reflected the altered behavior effecting medication adherence and employment status. Similar to Guillevin et al., patients reported significant dissatisfaction with their social well-being and financial hardship [3]. Physical limitations persisted despite most patients being on combination PAH therapy. Likewise, the financial burden existed despite the majority of patients (96.2%) having insurance with either private insurance or Medicare. Importantly, in the present study, employment declined by more than half from time of diagnosis and many patients applied for financial assistance and disability.

Interestingly, novel results in this study included questions that revealed targeted cost-coping strategies by patients that may result in suboptimal therapy. There was a concerning number of patients who skipped medication doses, delayed prescription, and procured medications from other countries. Those unconventional strategies potentially adversely affect treatment efficacy. That was not directly linked in this study; however, it is important for providers to be aware that patient’s behavior may be influenced by the cost of treatment.

The findings of this study were consistent with previous studies showing symptomatic effect on patients’ lives despite adequate PAH therapy. This study further documented an impact on employment that presumably compounded the financial burden of disease. As a consequence, many patients applied for financial assistance or disability.

There were several limitations to this study that included relatively small numbers in US patients and a methodology that has an inherent questionnaire bias. The questionnaire was designed to be descriptive but was not validated to assess the psychosocial and financial implications on PAH. Additionally, the majority of patients filled out their questionnaire in front of the proctor, potentially resulting in patient-perceived “favorable” answers. The MCF patients were consecutive patients from a single center while the PHA patients were those motivated and with the means to attend the PHA national meeting, both with potential selection bias that drove the statistical differences in the two cohorts. There were minor demographic differences between the two groups, but specific responses were not different between the two groups, so likely did not confound the results. Nonetheless, the general trends indicated ongoing challenges of managing the disease state and associated treatment, as well as, specific coping strategies.

In conclusion, a questionnaire assessment of the psychosocial, employment, and financial burden of PAH demonstrated a significant burden of disease despite medical treatment. In addition, the significant financial implications may have led to a volitional reduction in medication adherence. No previous studies have shown what the cost of PAH does to the therapy regimen of each individual patient. While further study is needed to see if the effect on patient specific therapy regimen alters outcomes, PAH patients require assistance to overcome the burdens identified in this study.

## Figures and Tables

**Table 1:** Patient demographics of the entire cohort and then separated into groups.

	All (*n* = 106)	MCF Patients (*n* = 57)	PHA Patients (*n* = 49)	*p* Value
Age	57.0 ± 13.6	61.9 ± 11.4	51.3 ± 13.8	<0.01
Gender, F	89 (84.0%)	46 (80.7%)	43 (87.8%)	0.43
Ethnicity				0.54
Not Hispanic or Latino	97 (91.5%)	53 (92.7%)	44 (89.8%)	
Hispanic or Latino	3 (2.8%)	2 (3.5%)	1 (2.0%)	
Unknown	6 (5.7%)	2 (3.5)	4 (8.2%)	
Race				0.36
White	97 (91.5%)	54 (94.7%)	43 (87.8%)	
Black	3 (2.8%)	2 (3.5%)	1 (2.0%)	
Asian	3 (2.8%)	1 (1.8%)	2 (4.1%)	
American Indian	1 (0.9%)	0	1 (0.9%)	
Unknown	2 (1.9%)	0	2 (1.9%)	
Home region				<0.01
Southeast	69 (65.1%)	53 (93.0%)	16 (32.7%)	
Southwest	5 (4.7%)	2 (3.5%)	3 (6.1%)	
Northeast	2 (1.9%)	0	2 (4.1%)	
Northwest	1 (0.9%)	0	1 (2.0%)	
Mid-west	11 (10.4%)	0	11 (22.5%)	
Mid-Atlantic	13 (12.3%)	2 (3.5%)	11 (22.5%)	
West	5 (4.7%)	0	5 (10.2%)	
PAH subgroup				<0.01
1.1	58 (54.7%)	23 (40.4%)	35 (71.4%)	
1.2	4 (3.8%)	0	4 (8.2%)	
1.3	2 (1.9%)	0	2 (4.1%)	
1.4.1	27 (25.5%)	21 (36.8%)	6 (12.2%)	
1.4.3	4 (3.8%)	4 (7.0%)	0	
1.4.4	11 (10.4%)	9 (15.8%)	2 (4.1%)	

The entire cohort was well mixed in age, PAH subgroup, and home region in the USA. MCF = Mayo Clinic Florida; PHA = Pulmonary Hypertension Association; F = female; PAH = pulmonary arterial hypertension. Continuous variables displayed as mean ± standard deviation and categorical variables displayed as number (percentage of total). Categorical data were compared using a Pearson χ^2^ or Fisher exact test, where appropriate depending on the number of patients in each analysis.

**Table 2:** Medication regimen for the cohort.

Medication	All (*n* = 106)	MCF (*n* = 57)	PHA (*n* = 49)	*p* Value
Phosphodiesterase-5 inhibitors				
Sildenafil	29 (27.4%)	17 (29.8%)	12 (24.5%)	0.54
Tadalafil	49 (46.2%)	23 (40.4%)	26 (53.1%)	0.19
Endothelin receptor antagonists				
Ambrisentan	38 (35.8%)	22 (38.6%)	16 (32.7%)	0.52
Bosentan	10 (9.4%)	6 (10.5%)	4 (8.2%)	0.75
Macitentan	31 (29.2%)	14 (24.6%)	17 (34.7%)	0.25
Soluble guanylate cyclase stimulant				
Riociguat	9 (8.5%)	2 (3.5%)	7 (14.3%)	0.08
Prostacyclin receptor agonist				
Selexipag	10 (9.4%)	4 (7.0%)	6 (12.2%)	0.51
Prostanoids				
Oral treprostinil	8 (7.5%)	2 (3.5%)	6 (12.2%)	0.14
Inhaled treprostinil	9 (8.5%)	7 (12.3%)	2 (4.1%)	0.17
Treprostinil infusion	24 (22.6%)	9 (15.8%)	15 (30.6%)	0.07
Epoprostenol infusion	10 (9.4%)	5 (8.8%)	5 (10.2%)	0.80
CCB add-on	9 (11.3%)	7 (12.3%)	2 (4.1%)	0.37
CCB monotherapy	3 (2.8%)	3 (5.3%)	0	0.55
Combination therapy (>1 medication)	80 (75.4%)	40 (70.2%)	40 (81.6%)	0.17

This table shows that treatment was adequate and similar regimens were used between the two cohorts. MCF = Mayo Clinic Florida; PHA = Pulmonary Hypertension Association; CCB = calcium channel blocker. Categorical variables displayed as number (percentage of total). Categorical data were compared using a Pearson χ^2^ or Fisher exact test, where appropriate depending on the number of patients in each analysis.

**Table 3:** Data on missed medication doses and appointments due to financial hardship.

	All (*n* = 106)
**How Often Do You Miss Medication Doses?**	
Never	77 (72.6%)
Once a month	11 (10.4%)
Once a week	6 (5.7%)
Daily	4 (3.8%)
I don’t know	8 (7.5%)
**Have You Had to Miss an Appointment?**	
Yes	11 (10.4%)

MCF = Mayo Clinic Florida; PHA = Pulmonary Hypertension Association. This table shows that nearly one in four patients miss medications and 1 in 10 miss appointments due to financial issues.

## Data Availability

Patient-level data available if requested.

## Supplementary Materials

The following are available online at https://www.mdpi.com/2079-9721/8/2/22/s1, Table S1: Questionnaire for evaluating the financial burden of PAH therapy in PH patients at MCF.
