# Supplementary material for: Psychosocial and Financial Burden of Therapy in USA Patients with Pulmonary Arterial Hypertension
Source: Diseases. 2020 Jun 13;8(2):22. doi: 10.3390/diseases8020022 (PMC7349780; doi:10.3390/diseases8020022)
Supplement: Supplementary file 1 [file diseases-08-00022-s001.pdf]

Questionnaire for evaluating the financial burden of PAH therapy in PH patients at MCF

**PULMONARY ARTERIAL HYPERTENSION SURVEILLANCE QUESTIONNAIRE**	
**Patient Name:**	**Date of Birth:**
**Gender:**	**Date of diagnosis :**

	**Yes:**	**No:**
**<u>Questionnaire:</u>**		
1. Are you NOW taking any medication prescribed by a doctor for your pulmonary hypertension?	☐	☐
If yes, which one?		
a) Revatio (Sildenafil)	☐	
b) Letairis (Ambrisentan)	☐	
c) Adempas (Riociguat)	☐	
d) Uptravi (Selexipag)	☐	
e) Tadalafil (Adcirca)	☐	
f) Orenitram (oral treprostinil)	☐	
g) Tyvaso (inhaled treprostinil)	☐	
h) Pump/Infusion Remodulin (Treprostinil)	☐	
i) Pump/Infusion Veletri (epoprostenol)	☐	
j) More than one medication	☐	
k) I don't remember	☐	
2. Do you have any symptoms despite taking your medication?	☐	☐
If yes, what are your symptoms?		
a) Shortness of breath	☐	
b) Palpitation (heart racing)	☐	
c) Chest pain	☐	
d) Lightheadedness with exertion(effort)	☐	
e) Ankle swelling	☐	
3. Now I am going to ask you about certain medical conditions. Have you EVER been told by a doctor or other health professional that you had one of the following:		
a) Heart attack	☐	
b) High blood sugar (Diabetes)	☐	
c) Hypertension, also called high blood pressure in your arm	☐	
d) Asthma	☐	
e) Chronic obstructive lung disease (emphysema)	☐	
f) Collagen vascular disease (like Scleroderma or Lupus)	☐	
g) Sleep apnea	☐	
h) Others: If yes ( )	☐	

4. Are you satisfied with your current level of social activity?

a) Not at all. ☐
b) A little bit ☐
c) Somewhat ☐
d) Quite a bit ☐
e) Very much ☐

5. Does your health now limit you in doing two hours of physical labor?
a) Not at all ☐
b) A little bit ☐
c) Somewhat ☐
d) Quite a bit ☐
e) Cannot do ☐

6. Does the medication affect your mood? ☐ ☐

7. Are you currently working? ☐ ☐

If no, were you employed when you were diagnosed and started treatment? ☐ ☐

8. How old were you when pulmonary hypertension was first diagnosed?

9. How many hours do you work per week? (escape if you aren't working)

a) Less than 10 hours per week ☐
b) More than 10 and less than 20 hours per week ☐
c) More than 20 and less than 30 hours per week ☐
d) More than 30 and less than 40 hours per week ☐
e) More than 40 hours per week ☐

10. How many days have you missed from work last year?

a) Less than 10 days per year. ☐
b) 10-20 days per year. ☐
c) 20-30 days per year. ☐
d) >30 days per year. ☐
e) I don't know ☐

11. Did you have health/medical insurance at the time of diagnosis? ☐ ☐

If yes, which one?

a) Private ☐
b) Medicare ☐
c) Medicaid ☐
d) Other ☐

12. Did you have health/medical insurance that covers your pharmacy cost at the time of diagnosis? ☐ ☐

If yes, which one?

e) Private ☐
f) Medicare ☐
g) Medicaid ☐
h) Other ☐

13. Did you have out of pocket expenses at that time? ☐ ☐

14. Does your health insurance still cover the expenses of pulmonary hypertension? ☐ ☐

15. Does your health insurance still cover the expenses of pulmonary hypertension medications? ☐ ☐

16. Do you feel that the out of pocket medications expenses are affordable?? ☐ ☐

17. Have you applied for disability? ☐ ☐

If yes, is it related to the pulmonary hypertension? ☐ ☐

18. What are your monthly out of pocket expenses at this time for PH therapy?

a) Less than $ 1000 per month. ☐
b) >$1000- <2000 per month ☐
c) >$2000- <3000 per month ☐
d) >$3000- <4000 per month ☐
e) More than $4000 per month ☐
f) I don't know ☐

19. DURING THE PAST 12 MONTHS, How have your out of pocket expenses changed with different medications?

a) Didn't change. ☐
b) Affected your daily lifestyle. ☐
c) I don't know ☐
d) Other ☐

20. DURING THE PAST 12 MONTHS, were any of the following true for you regarding your pulmonary hypertension medications?
a) You took less medicine to save money. ☐
b) You skipped medication doses to save money. ☐
c) You delayed filling a prescription to save money. ☐
d) You asked your doctor for a lower cost medication to save money. ☐
e) You bought prescription drugs from another country to save money. ☐
f) You used alternative therapies to save money. ☐

21. Were you forced to change your medication because of the financial hardship? ☐ ☐

22. How frequently did you miss the drug dosages?

a) I don't miss any drug dosages. ☐
b) Daily ☐
c) Once per week ☐
d) Once per month ☐
e) I don't know ☐

23. Do you have additional financial support for the expenses of pulmonary hypertension? ☐ ☐

If yes, from whom?
a) Nonprofit organization (like Caring Voice Coalition) ☐
b) Pharmaceutical company assistance program
c) Relatives/Friends ☐
d) Other ☐

24. Were there any time when you needed a follow-up care, but didn't get it because you couldn't afford it?

a) Yes ☐
b) No ☐
c) Don't know ☐
d) Refused ☐

25. In regard to your health insurance or health care coverage, how does it compare to a year ago?
a. Better ☐
b) Worse ☐
c) About the same ☐
d) Don't know ☐
e) Refused ☐

**Name of patient:**	**Date:**
**Signature of investigator :**	**Date:**
